# New method to apply the lumbar lordosis of standing radiographs to supine CT-based virtual 3D lumbar spine models

**DOI:** 10.1038/s41598-022-24570-2

**Published:** 2022-11-27

**Authors:** Benjamin Hajnal, Peter Endre Eltes, Ferenc Bereczki, Mate Turbucz, Jennifer Fayad, Agoston Jakab Pokorni, Aron Lazary

**Affiliations:** 1 In Silico Biomechanics Laboratory, National Center for Spinal Disorders, Buda Health Center, Királyhágó St. 1-3, Budapest, 1126 Hungary; 2 School of PhD Studies, Semmelweis University, Budapest, Hungary; 3 Department of Spine Surgery, Department of Orthopaedics, Semmelweis University, Budapest, Hungary; 4 Department of Industrial Engineering, Alma Mater Studiorum, Universita Di Bologna, Bologna, Italy; 5 National Center for Spinal Disorders, Buda Health Center, Budapest, Hungary

**Keywords:** Biomedical engineering, Three-dimensional imaging, Computational science

## Abstract

Standing radiographs play an important role in the characterization of spinal sagittal alignment, as they depict the spine under physiologic loading conditions. However, there is no commonly available method to apply the lumbar lordosis of standing radiographs to supine CT-based virtual 3D models of the lumbar spine. We aimed to develop a method for the sagittal rigid-body registration of vertebrae to standing radiographs, using the exact geometry reconstructed from CT-data. In a cohort of 50 patients with monosegmental spinal degeneration, segmentation and registration of the lumbar vertebrae and sacrum were performed by two independent investigators. Intersegmental angles and lumbar lordosis were measured both in CT scans and radiographs. Vertebrae were registered using the X-ray module of Materialise Mimics software. Postregistrational midsagittal sections were constructed of the sagittal midplane sections of the registered 3D lumbar spine geometries. Mean Hausdorff distance was measured between corresponding registered vertebral geometries. The registration process minimized the difference between the X-rays’ and postregistrational midsagittal sections’ lordoses. Intra- and inter-rater reliability was excellent based on angle and mean Hausdorff distance measurements. We propose an accessible, accurate, and reproducible method for creating patient-specific 3D geometries of the lumbar spine that accurately represent spinal sagittal alignment in the standing position.

## Introduction

There are a growing number of in silico studies that use finite element modeling (FEM) to address spinal physiology and pathology^1–4^. Various aspects that have been studied include behavior under different loading conditions^5^, effects of spinal pathologies, and surgical solutions^6,7^. One of the limitations of these studies is that the used patient-specific 3D spine geometries are usually obtained by the segmentation of CT (computed tomography) scans. These usually depict the spine in the supine position, as opposed to the upright position of radiographs which reflect spinal alignment under physiologic loading conditions and are routinely used to measure pelvic parameters. Pelvic parameters and especially lumbar lordosis (LL) have great significance in clinical evaluation, surgical planning, and post-operative outcome of spinal disorders^6,8^. Naserkhaki^9^ and his collegues in 2016 demonstrated in a finite element method-based study that the curvatures of the lumbar spine strongly influenced the magnitude and location of loads on the spinal components and also altered the kinematics and load-sharing, particularly in extension. Based on his study results, Naserkhaki concluded that subject-specific geometry and sagittal curvature should be an integral part of the mechanical analysis of the lumbar spine.

The sagittal geometry of the spine depends on the position of the patient, therefore radiographic measurements cannot be equated between different imaging modalities^10–14^. The differences between the supine and upright positions of the spine have been studied in several patient groups, including patients with spinal trauma and spinal deformity^14–19^. These studies also highlight the clinical relevance of using standing spine radiographs in addition to supine imaging.

Several methods have been proposed both for 3D-2D registration of the spine (manual and automatic)^20,21^ as well as inference-based models for the reconstruction of 3D spinal anatomy from standing biplanar radiographs^22–24^. Published registration and reconstruction-based methods can take into account the sagittal as well as coronal alignment of the spine and are able to accurately reproduce patient-specific vertebral geometry. Some of these leverage EOS® imaging which is shown to be highly accurate^25^ but has limited clinical availability due to its high cost. None of these methods directly uses CT-derived vertebral geometry, which has the benefit of exceptional geometric fidelity and potential for QCT-based (quantitative CT) material property assignment, from which FEM-based simulations could benefit. A common limitation of most published methods is that they employ in-house scripts and other software that are not publicly available and require a great amount of technical knowledge to use.

Based on these observations, we aimed to develop an accessible, accurate, and reproducible method, using a commercially available software solution, which has published uses in the field of orthopedics, for the rigid-body registration of the exact 3D vertebral geometries obtained by the segmentation of CT scans, to the corresponding biplanar standing spine radiographs for further use in in silico clinical studies and to facilitate the deep learning-based automation of the same process by building a suitable database from the resulting geometries. Segmentation and registration of these data allows for the creation of a highly accurate and patient-specific, standing 3D model of the spine which can be used in in silico clinical studies. Our focus is on the sagittal alignment of the lumbar spine, due to its clinical relevance and the high availability of CT imaging data of this region.

## Materials and methods

### Cohort

Retrospective CT and radiographic data from a cohort of 50 patients were used for this study. The original database was created by the National Center for Spinal Disorders in the scope of the MySpine (FP7 HEALTH-F2-2008–269,909) international research project, involving 250 patients with monosegmental spinal degeneration as an inclusion criterion. Out of these, 50 was selected showing no signs of severe coronal plane malalignment of the lumbar spine. Biplanar X-ray images were taken with the ddRAura™ OTC- Swissray, direct digital X-Ray system, while the CT-scans were made with a Hitachi Presto CT machine with a voltage of 120 kV and intensity of 225 mA (protocol was defined in the MySpine project). Reconstruction was done with a voxel size of 0.6 × 0.6 × 0.6 mm^3^. The study involving human participants were reviewed and approved by the National Ethics Committee of Hungary and the National Institute of Pharmacy and Nutrition (reference number: OGYÉI/163–4/2019). Informed consent was obtained from the participants. The participants provided their written informed consent to participate in this study. All methods were carried out in accordance with relevant guidelines and regulations.

The data were exported from the hospital’s PACS into DICOM file format. To comply with the ethical approval and the patient data protection, anonymization of the DICOM data was performed using the freely available Clinical Trial Processor software (Radiological Society of North America, https://www.rsna.org/ctp.aspx)26. The study was approved by the National Ethics Committee of Hungary, the National Institute of Pharmacy and Nutrition (reference number: OGYÉI/163–4/2019).

### Segmentation and creation of 3D vertebral geometries

3D geometries of the lumbar vertebrae (L1-L5) and the sacrum (S) were acquired by segmenting the CT scans with Mimics® (Mimics Research, Mimics Innovation Suite 21.0, Materialise, Leuven, Belgium) software. The segmentation process was performed by two independent investigators (I_1_, I_2_), for the evaluation of segmentation accuracy. The results of the two sets of segmentations were compared using the Dice similarity index ($DSI = \frac{{2 \times \left| {X \cap Y} \right|}}{\left| X \right| + \left| Y \right|}$, where X and Y are the compared sets, DSI can range between 0 and 1, higher values indicating more overlap between sets).

**Figure 1:**
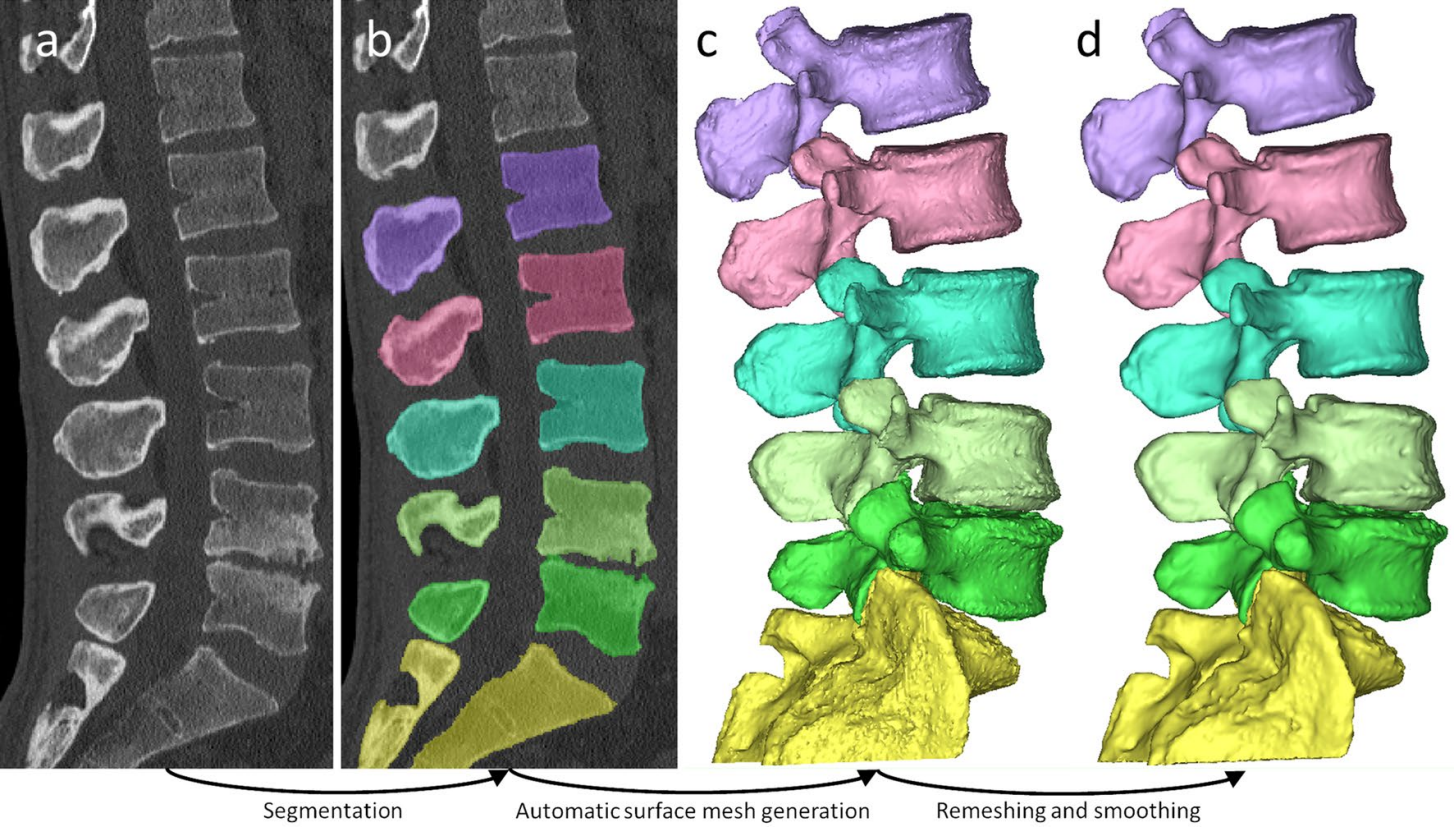
Creation of CT-based 3D geometry. (**a**) CT scan (**b**) segmentation masks (**c**) automatically generated triangle-based surface mesh (**d**) triangle mesh after uniform remeshing and smoothing.

Surface triangle meshes were generated from the resulting masks, which were uniformly remeshed and smoothed (Fig. 1). See Supplementary Method 1 for the used post-processing protocol.

### Intersegmental and LL angle measurement

**Figure 2:**
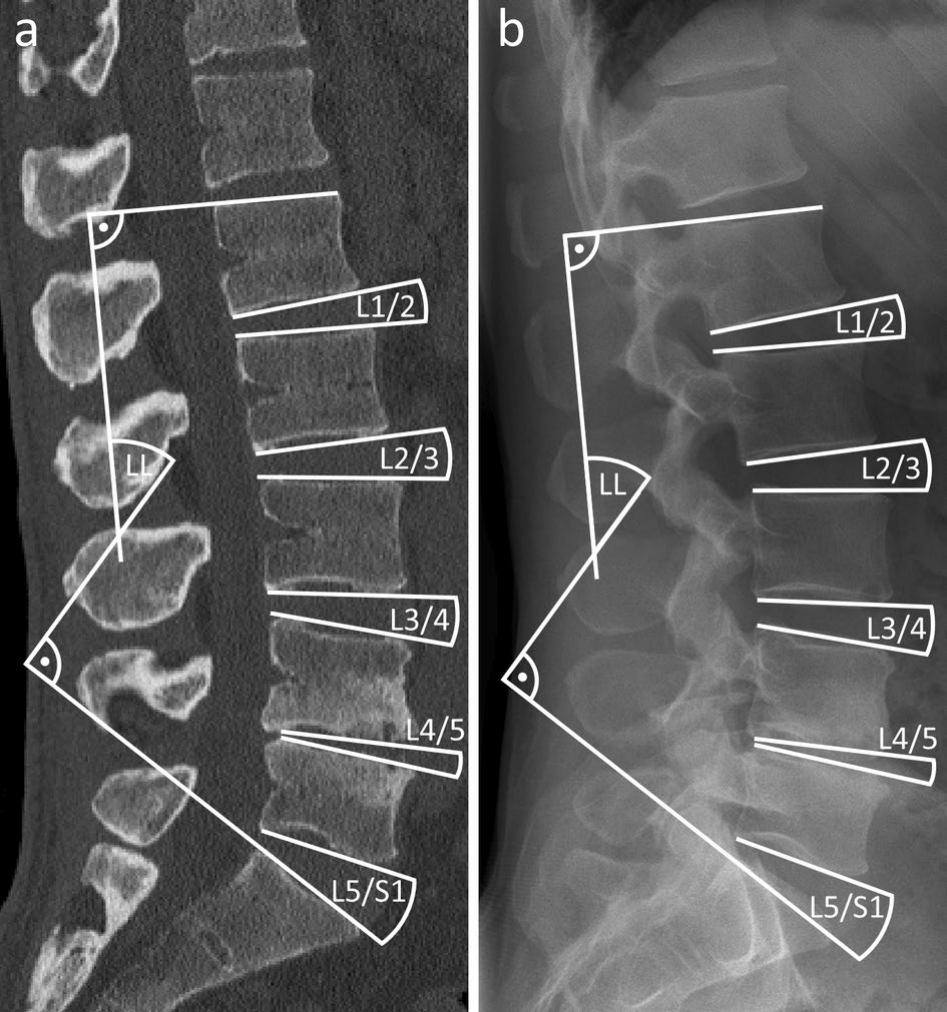
Measurement of intersegmental and LL angles in a (**a**) CT-scan and (**b**) radiograph.

The LL (between the cranial endplate of L1 and the cranial endplate of S1) and intersegmental angles were digitally measured in the sagittal radiographs and CT scans between each segment using Surgimap® software (Nemaris Inc, New York, NY, USA) (Fig. 2). All measurements were performed by two independent investigators (I_1,_ I_2_) at two points in time (T_1,_ T_2_). For CT scans, the sagittal midplane between the left and right vertebral margins was used for measurement.

### Registration

**Figure 3:**
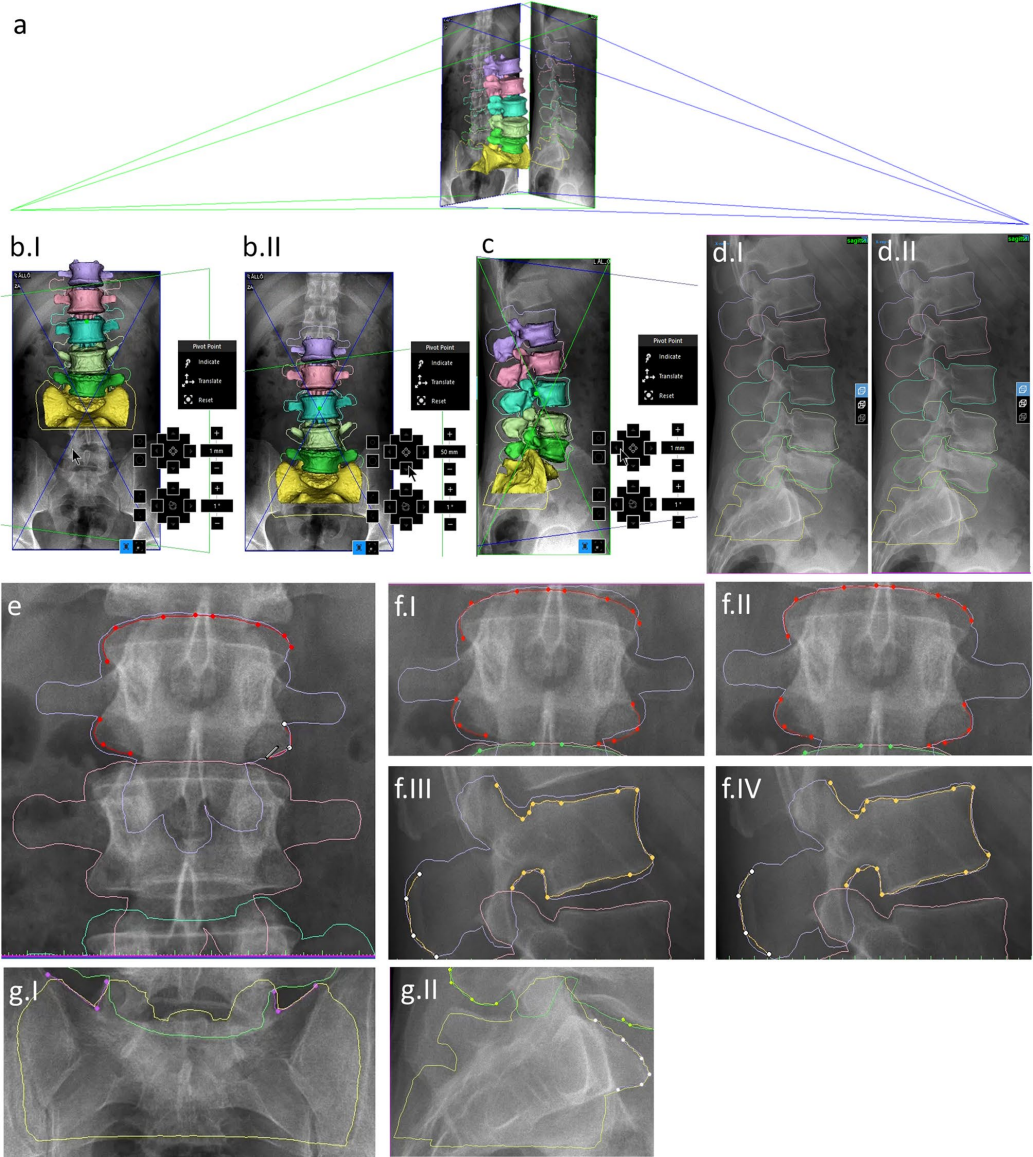
Registration of 3D lumbar spine geometry to standing radiographs. **(a)** simulated 3D environment with 3D lumbar spine geometry, radiographs, and radiation sources. (**b**.I, II) manual registration of X-rays, before and after. (**c**) manual registration of vertebrae, 3D viewfinder with manual registration toolbar present. (**d.**I, II) manual registration of vertebrae, before and after **(e)** contour selection, using the contouring tool. (**f**) automatic contour-based registration of a vertebra in the (**f**.I, II) frontal and (**f**.III, IV) sagittal planes before and after. (**g**) contours used for the registration of the sacrum. (Mimics® 21.0, materialise.com).

The rigid registration of 3D vertebral geometries to the respective standing spine radiographs was done by two investigators (I_1,_ I_2_) independently at two points in time (T_1,_ T_2_), using the X-ray module of Mimics® software. The software allows the user to align 3D geometries to planar images based on projected contours, which are generated based on a simulated 3D environment that includes the 3D geometries, planar images and radiation sources (Fig. 3a). Our method for registration consists of four distinct steps. These are the following: 1) manual registration of X-rays, 2) manual registration of vertebrae, 3) contour selection, and 4) automatic contour-based registration.

Following the import of X-ray images from a DICOM file, the X-rays are placed in the simulated environment, orthogonal to each other, with correctly simulated radiation sources for contour projection and correct orientation and scale respective to the 3D geometries as these data are embedded in the DICOM file (if not, manual adjustments can be made). However, they still need to be adjusted with translational operations mostly along the z-axis. This is done by manual X-ray registration, based on projected contours in both planes (Fig. 3b). This step was only done once for each case (at I_1_T_1_) to ensure that the geometries resulting from independent registrations are in the same coordinate system for the calculation of Hausdorff distances (see later).

Manual registration of vertebrae is also based on projected contours. Individual vertebrae are aligned to the X-ray images in both planes, which can be simultaneously monitored through separate viewports. Translational and rotational operations are both permitted during this step. All vertebrae and the sacrum are aligned in this way (Fig. 3c and d).

Contours are selected in both X-ray images for each vertebra and the sacrum. The contouring tool for this purpose allows the creation of contours based on control points. Between the control points, the contour line is ‘attracted’ to gradients in its neighborhood, thus facilitating quick and precise contour selection. If the edge is not detected automatically, straight lines and splines can also be drawn (Fig. 3e). Several contour sections can be added to a contour object, which ought to consist of well-defined edges of the vertebrae on the radiograph. In the case of lumbar vertebrae these are usually the projected edges of the corpus and the proximal aspects of the transverse processes in the frontal plane, as well as the projected edges of the corpus, proximal aspects of articular processes and dorsal ridge of the spinous process in the sagittal plane (Fig. 3f). In the case of the sacrum this is usually the projected edge of the sacral wing in the frontal plane and the projected edge of promontory in the sagittal plane (Fig. 3g). These are the regions mainly used for contouring during the registration process.

Contours selected in the previous step allow for automatic contour-based registration of the individual 3D vertebral geometries. During contour-based registration, the projected contours of 3D geometries are automatically aligned to the selected target contour objects by the slight adjustment of the 3D object’s position (Fig. 3f).

After these steps, the correctly aligned vertebral geometries were exported as STL files.

See Supplementary Method 2 for the used registration protocol.

### Postregistrational midsagittal sections

**Figure 4:**
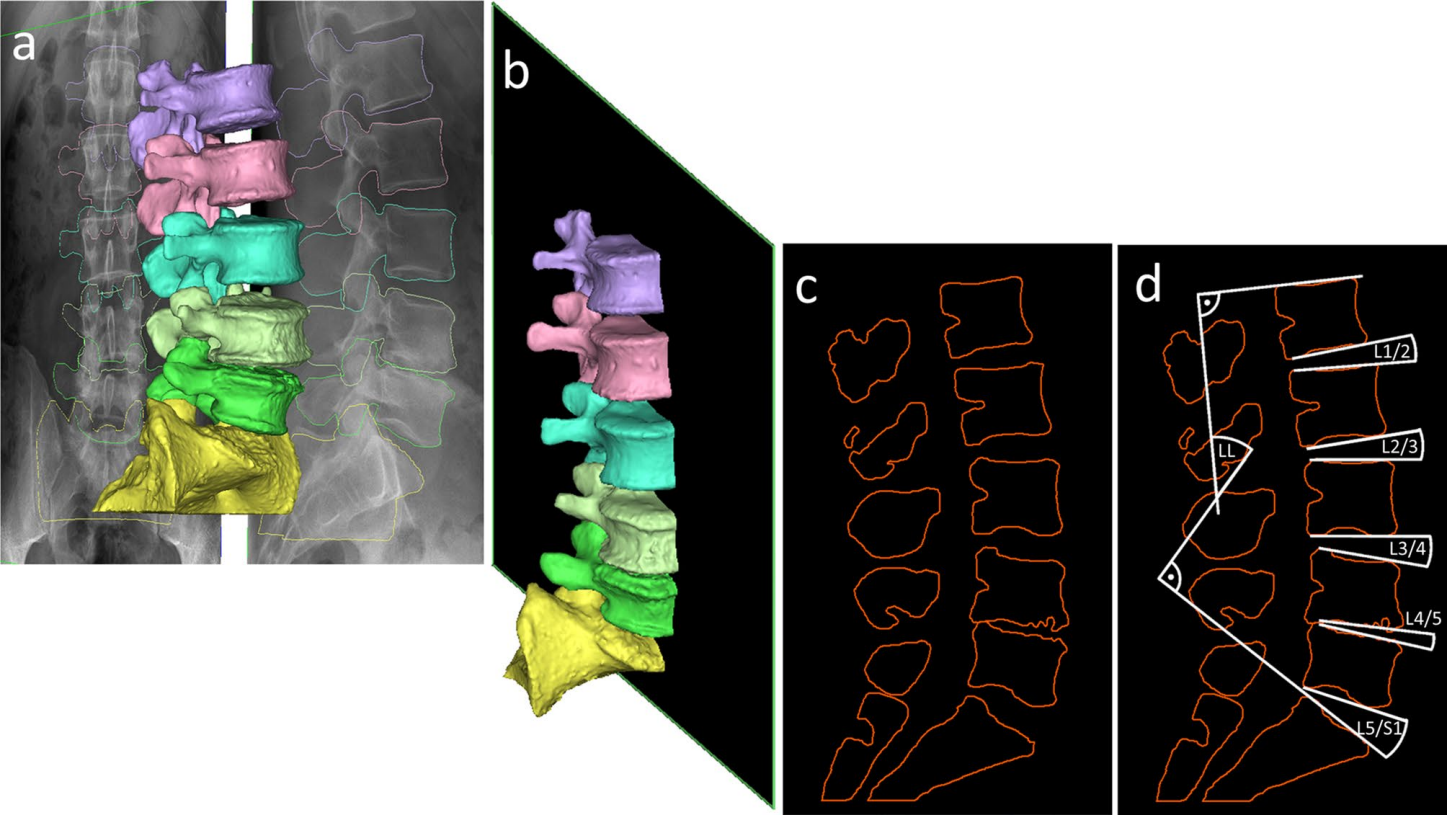
Creation of postregistrational midsagittal sections. (**a**) registered 3D lumbar spine geometry. (**b**) mid-plane used for sectioning. (**c**) resulting postregistrational midsagittal section image. (**d**) intersegmental and LL angle measurements in the postregistrational midsagittal section.

By making a sagittal mid-plane section between the left and right vertebral edges of the registered geometry, postregistrational midsagittal sections have been made for each patient within Mimics® software (Fig. 4). These postregistrational midsagittal sections are analogous to the sections made from the scans for angle measurement, thus suitable for comparison with other modalities regarding intersegmental and LL angles.

### Accuracy and reproducibility

Registration accuracy has been quantified by comparing postregistrational midsagittal sections with the respective standing radiographs, based on intersegmental and LL angle measurements.

Every major step in the presented method has been tested for reproducibility by being carried out by the two independent investigators.

**Figure 5:**
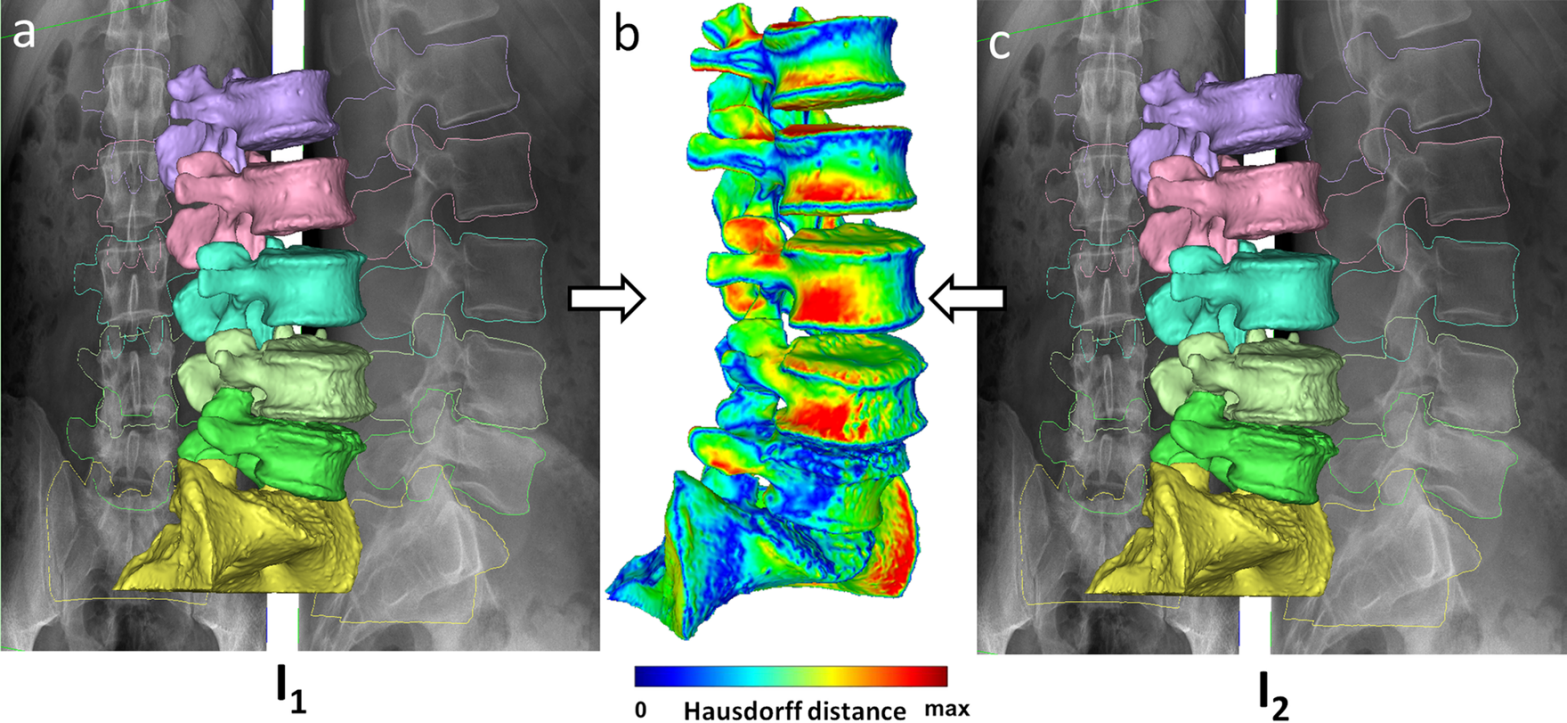
Spatial distribution of the distance of identical meshes between independent registrations. (**a** and **c**) 3D views of lumbar spine geometry registered by I_1_ and I_2_ respectively. (**b**) 3D heatmap of the spatial distribution of distances between the two meshes.

The results of the two sets of registrations were compared twofold. Once based on intersegmental and LL angle measurements and once based on the MeshLab^27^ implementation of the Metro algorithm^28^ for comparing 3D surface meshes (Fig. 5). The mean distance was used to measure the difference between the surface meshes of the corresponding segments, which is defined as the surface integral of the distance divided by the area ($E_{m} \left( {S_{1} ,S_{2} } \right) = \frac{1}{{\left| {S_{1} } \right|}}\mathop \smallint \limits_{{S_{1} }}^{ } e\left( {p,S_{2} } \right) ds$., where S_1_ and S_2_ are the surfaces being compared, p is a point of S_1_ and e is the distance of a point and a surface).

The results of the two sets of angle measurements were compared using intraclass correlation coefficients.

**Figure 6:**
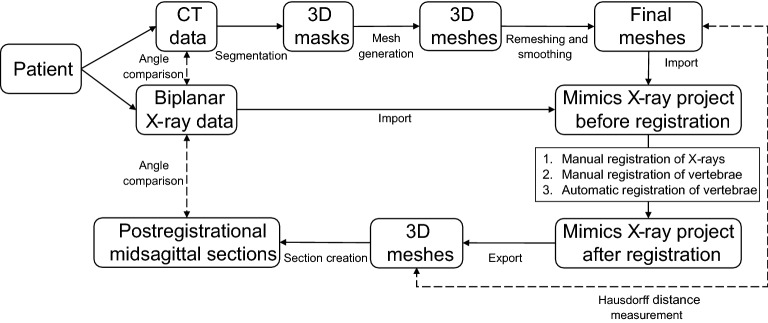
Flowchart of the used methods, with measurements denoted as dashed double-headed arrows.

See Fig. 6 for a concise overview of the methods in flowchart format.

### Statistical analysis

All statistical tests were performed with SPSS® Statistics 25.0 (IBM® Corp., Armonk, NY, USA).

Inter-rater (I_1_ vs I_2_) reliability was determined by Intraclass Correlation Coefficient (ICC) estimates and their 95% confidence intervals (CI) were calculated based on a mean-rating (k = 2), absolute-agreement, 2-way mixed-effects model. Intra-rater (I_1_T_1_ vs I_1_T_2_, I_2_T_1_ vs I_2_T_2_) reliability was determined by ICC estimates and their 95% confidence intervals were calculated based on a single measurement, absolute-agreement, 2-way mixed-effects model.

## Results

### Segmentation accuracy

The calculated DSIs were > 0.9 in every case, which denotes a high overlap between the segmentations of the two investigators, meaning excellent segmentation accuracy. See Supplementary Table 1 for measurements.

### Angle measurement accuracy

ICCs for each intersegmental angle and LL were > 0.8 measured on CT scan, X-ray and postregistrational midsagittal section, which means excellent reliability for the intra- and inter-rater tests^29^. See Supplementary Table 2, 3, and 4 for intra-class correlations of X-ray, CT, and postregistrational midsagittal section measurements, respectively and Supplementary Dataset 1 for measurements.

### Angle differences between supine and upright alignments

Intersegmental and LL angles were compared for every segment between CT scans and standing spine radiographs, which showed an average absolute difference of 2–3.4° between corresponding segments, and 5.6° between LLs. The average signed differences found, were similar to the findings of Benditz et al^30^.

### Registration accuracy

**Figure 7:**
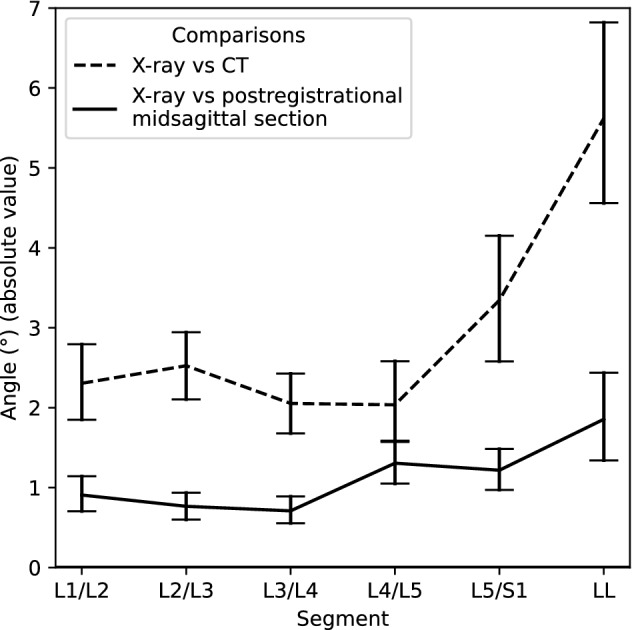
Average absolute value differences in degrees between intersegmental and LL angles measured in postregistrational midsagittal sections and standing radiographs, compared to the previous measurement (between X-rays and CTs). Error bars indicate 95% confidence intervals.

The average absolute difference between corresponding intersegmental and LL angles measured in postregistrational midsagittal sections and standing radiographs were < 1.3° and 1.9° respectively (Fig. 7).

**Figure 8:**
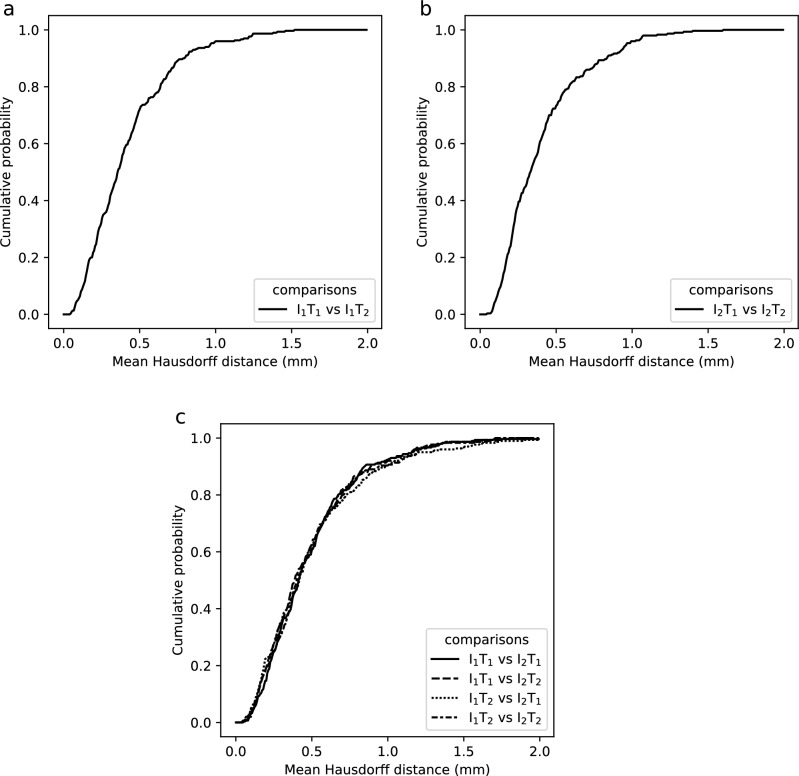
Registration accuracy measurements based on the distance between identical meshes from different registrations. The diagrams show the cumulative probabilities of mean distances between meshes for each comparison. (**a**) and (**b**) shows the intra-rater comparison of I_1_ (I_1_T_1_ vs I_1_T_2_) and I_2_ (I_2_T_1_ vs I_2_T_2_) respectively. (**c**) shows the inter-rater comparisons.

In intra-rater and inter-rater comparisons, the mean distance of the surface meshes was < 1 mm for ~ 96% and ~ 93% of the cases, respectively (Fig. 8). See Supplementary Dataset 2 for measurements.

## Discussion

In the present study we investigated the accuracy and viability of a novel method to apply the lumbar lordosis of standing radiographs to supine CT-based virtual 3D lumbar spine models. The method leverages the capabilities of the commercially available X-ray module of Materialise Mimics® software for the rigid registration of lumbar vertebral 3D geometries to biplanar standing radiographs. In their 2019 paper^31^, Pieroh et al. successfully used the same software package for the evaluation of sacroiliac screw loosening as well as several others in different orthopedic use cases^32–35^.

Although there are examples of both manual^20^ and automatic^10,21,24^ registration-based methods, a common limitation of those is that they employ in-house scripts and other software that are not publicly available and require a great amount of technical knowledge to use. Our method utilises commercially available software which allows the accurate 3D-2D registration of vertebrae. We aimed to show that the developed method is reasonably accurate (registration error is within the limits of variation within a person’s standing posture) and therefore has the potential to improve the accuracy of finite element analyses, used both in in silico clinical studies and certain clinical settings. To this end, we chose several different ways of measuring segmentation and registration accuracy in a cohort of 50 patients with monosegmental degeneration and without severe coronal malalignment of the lumbar spine. The cohort inclusion criteria were chosen in order to somewhat isolate the effect of upright position on lumbar lordosis which was the main focus of our study and covers most clinical cases, hence we have only investigated separately the accuracy of sagittal alignment. However, the mean Hausdorff distance measurements indirectly show that the registration is accurate in all planes. Segmentation, registration, and manual angle measurement were each done by two independent investigators, the latter two at two different time points. This allowed the calculation of both intra- and inter-rater agreements. Based on these measurements, the new method is reasonably accurate for in silico studies to benefit from the resulting geometries^9^. Whereas other published methods are based on parametric 3D-2D registration or inference based, our method is the first to combine the highly accurate 3D geometries derived from CT imaging with the spatial information that can be aqcuired by biplanar radiography.

We would like to highlight the limitations of our study. A minor limitation of our method is the time needed for registration, which can take up to an hour depending on the experience of the user, spinal geometry, and quality of the available radiographs. The most time-consuming parts being the manual registration and selection of contours. Using less control points and rougher manual registration can speed up the process, by sacrificing some of the accuracy. The manual segmentation of the lumbar spine, which is needed for the presented technique, is also labor intensive. This underlines the need for a similar but, ideally, fully automated method to increase the viability of 3D alignment correction in clinical practice. Another limitation is due to the technique of X-ray acquisition. If the radiographs are not taken simultaneously (e.g. by an EOS® device), small differences in posture between the frontal and sagittal radiographs can occur. If present, these differences can cause some degree of ambiguity during the registration process. Although limited discrepancies which are consistent with the physiological standing position should result in intermediary, biomechanically valid models of the standing spine, care should be taken to keep the patient’s position during the acquisition of radiographs.

## Conclusions

The proposed method offers an accessible, accurate and reproducible way of producing patient-specific 3D lumbar spine geometries that represent the standing alignment of the spine. A notable advantage of this technique is the utilization of commercially available software which may also be used in a clinical setting.

### Possible future applications

The results acquired in the process of making this paper led to the accumulation of new data that can be used for the development of deep learning-based algorithms to automate the registration process. This could be integrated in a third-party software solution, especially Mimics, due to its capability for workflow automation by scripting (Python)^36–38^. Additionally, the proposed method can be improved upon by implementing currently published but commercially unavailable automation techniques for spine segmentation^39^ and inferring the 3D geometry of the spine from standing biplanar radiographs^24^.

The presented method could have potential use cases in personalized medicine, complementing the currently used FEM-based techniques of surgical planning^40–42^. Our method can be used with already available radiologic data and has a steep learning curve.

Another potential use case after further research and validation could be in in silico studies to leverage the information in retrospective patient data by combining data from CT images with data from X-ray images.

## Supplementary Information

Supplementary Information 1.

Supplementary Information 2.

Supplementary Information 3.

## Data Availability

The original contributions presented in the study are included in the article/Supplementary Material; further inquiries can be directed to the corresponding author.
