# Supplementary material for: New method to apply the lumbar lordosis of standing radiographs to supine CT-based virtual 3D lumbar spine models
Source: Sci Rep. 2022 Nov 27;12:20382. doi: 10.1038/s41598-022-24570-2 (PMC9701766; doi:10.1038/s41598-022-24570-2)
Supplement: Supplementary file 3 — Supplementary Information 3. [file 41598_2022_24570_MOESM3_ESM.pdf]

New method to apply the lumbar lordosis of standing radiographs to supine CT-based virtual 3D lumbar spine models

Supplementary material

Benjamin Hajnal[1,2*], Peter Endre Eltes[1,3*], Ferenc Bereczki[1,2], Mate Turbucz[1,2], Jennifer Fayad[1,4], Agoston Jakab Pokorni[1], Aron Lazary[3,5]

1. In Silico Biomechanics Laboratory, National Center for Spinal Disorders, Buda Health Center, Budapest, Hungary
2. School of PhD Studies, Semmelweis University, Budapest
3. Department of Spine Surgery, Department of Orthopaedics, Semmelweis University, Budapest, Hungary
4. Department of Industrial Engineering, Alma Mater Studiorum, Universita di Bologna, Bologna, Italy
5. National Center for Spinal Disorders, Buda Health Center, Budapest, Hungary

*authors contributed equally to the work

Peter Endre Eltes, corresponding author
National Center for Spinal Disorders, Királyhágó St. 1-3, Budapest 1126, Hungary
Tel.:(36) 1-887-7900, Fax.: (36) 1-887-7987, Email address: eltespeter@yahoo.com

Supplementary method 1: Post-processing protocol for 50 patient cohort study

This protocol was written, using Mimics Research (version 21.0, Materialise, Leuven, Belgium) and Materialise 3-matic Research (version 13.0, Materialise, Leuven, Belgium). The aim is to create smoothed, uniformly remeshed vertebral and sacral geometries.

1. Make a working directory, named the last three digits of patient code. Put the respective .mcs into it.
2. Make the masks solid:
   a. Use the *segment* > *smart fill* tool with 1 voxel hole closing distance to fill the holes, then always check for remaining cavities throughout the mask
   b. If there is a bigger hole, you can use the *mark hole* option with appropriate parameters
   c. In the case of the sacrum:
      i. if it is cut through at the bottom, you should fill in the last slice with the *segment* > *edit masks* and *segment* > *cavity fill* (without multiple layer) tools, as it will be regarded as a huge hole
      ii. in other parts of the sacrum, there will be huge holes which will have to be manually patched (for me, this is the most time-consuming part of the whole process)
3. Remove outliers:
   a. Especially in the case of the corpuses of vertebrae there are usually spikes. You can use several different tool to eliminate them, is *segment* > *morphology operations* > *open*. Unfortunately, you can only do this one mask at a time.

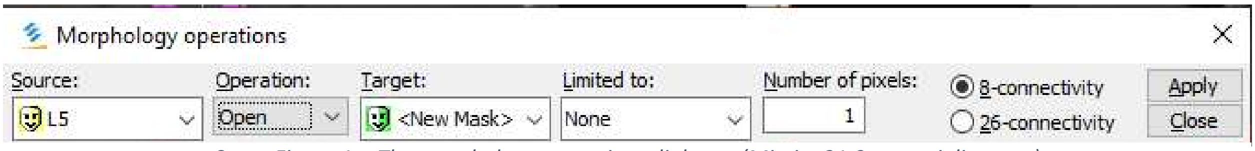

Supp. Figure 1 The morphology operations dialogue (Mimics 21.0, materialise.com)

4. Making parts:
   a. Select all final masks and right click to *calculate part* with *optimal* quality:
      i. if this gives you an error message, make sure that your mask is one piece with *segment* > *region grow*
   b. Smooth them with the following parameters:
      i. iterations: 6
      ii. smooth factor: 0.7
      iii. with shrinkage compensation
   c. Name your L1 to S masks 1 to 6 respectively
   d. Select masks 1 to 6 and in the right click menu choose *remesh*
5. Remesh parts:
   a. In 3-matic apply *remesh* > *uniform remesh* with the following parameters:
      i. entities: 1 to 6
      ii. target triangle edge length: 0.6
      iii. with sharp edge preservation
      iv. sharp edge angle: 60
      v. (autofix is ticked and no. of iterations is 3)
   b. Export STLs with *file* > *export* > *STL*:
      i. select entities 1 to 6
      ii. set the output directory to your working directory and apply
   c. Save .mxp source file with the working directory's name in the working directory

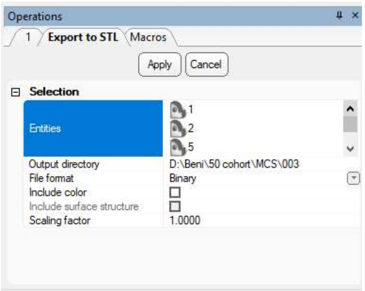

Supp. Figure 2 STL export dialogue (Mimics 21.0, materialise.com)

Supplementary method 2: Mimics X-ray Module Spine Registration Protocol

This protocol was written, using Mimics Research (version 21.0, Materialise, Leuven, Belgium). The aim is the rigid, contour-based registration of vertebral and sacral 3D geometries to standing, biplanar radiographs.

You should start out by opening an existing .mcs project file. It should contain the STL files you want to align with the X-ray images. Consider making copies of your 3D objects before changing their positions.

1. Opening the X-ray images:
   a. In the *X-RAY* tab choose the *Import X-ray* option (*X-RAY* > *Import X-ray* for short)

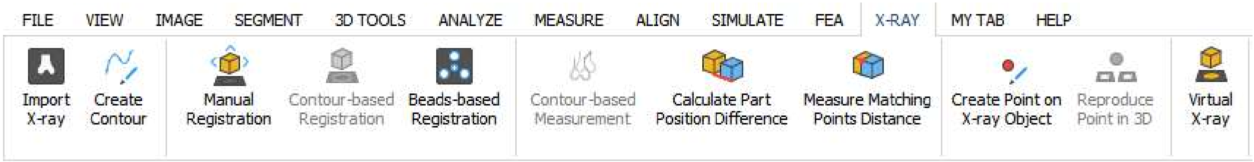

Supp. Figure 3 The X-RAY tab (Mimics 21.0, materialise.com)

   b. Select the desired coronal and saggital X-rays (select multiple files with the SHIFT or CTRL modifiers)
   c. After clicking *Next* and *Open*, the X-rays are showing in you viewport

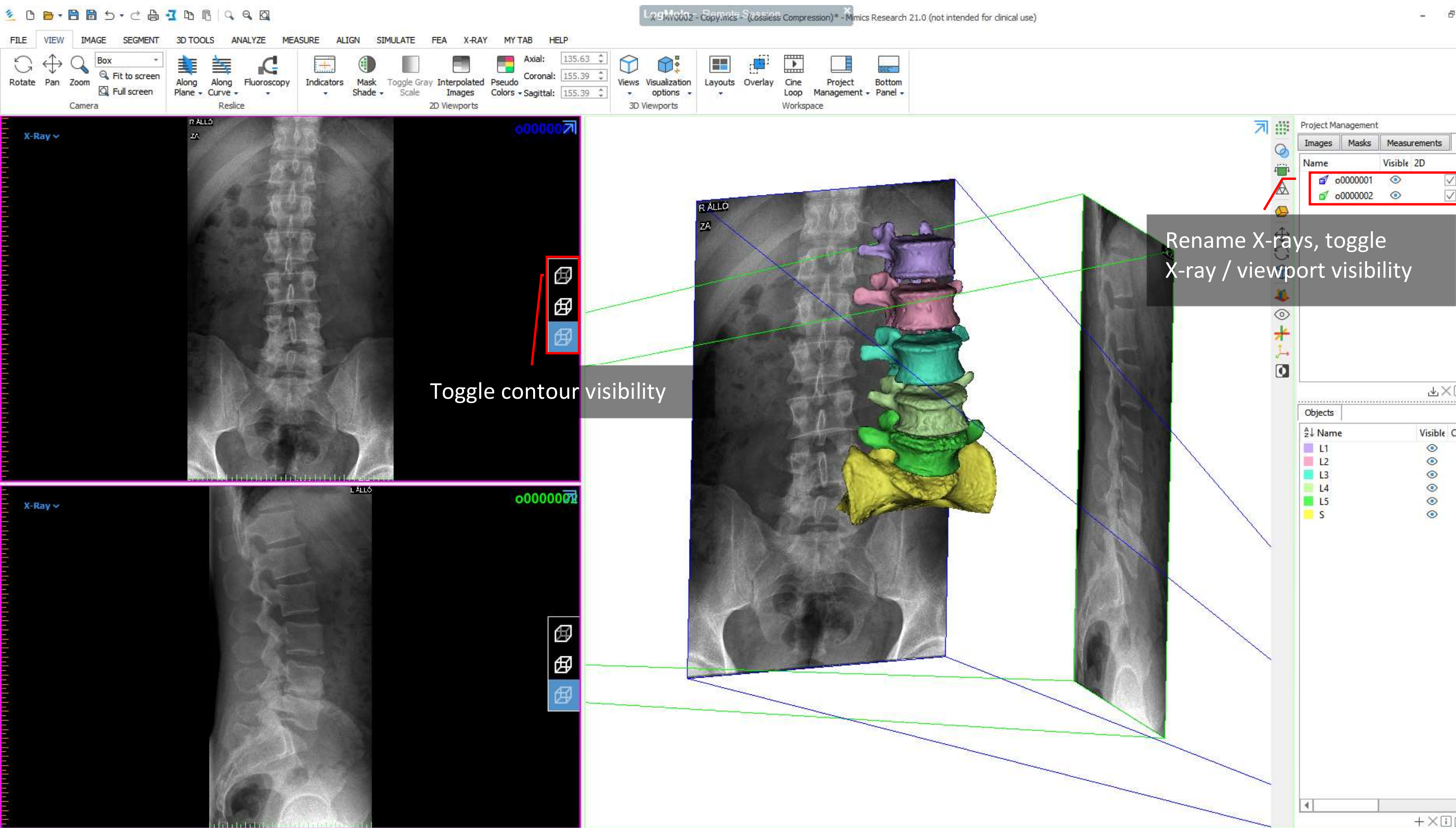

Supp. Figure 4 Mimics window after inserting the X-rays (Mimics 21.0, materialise.com)

2. Manual registration:
   a. As you can see, the X-rays are not aligned with the 3D objects. You have to use *X-RAY* > *Manual registration* to align all objects with the X-rays.
   b. In the pop-up dialog you will have to choose 3 parameters:
      i. *Type* of registration
      ii. Moving entity

iii. *Fixed entity*

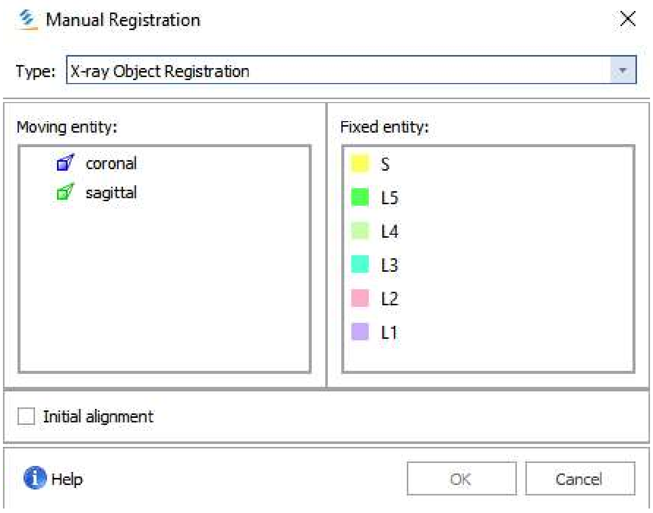

Supp. Figure 5 Manual registration dialog (I have renamed my X-rays as 'coronal' and 'sagittal') (Mimics 21.0, materialise.com)

iv. (*Initial alignment* should be unchecked)

c. Registration of the X-rays:
   i. Toggle on visibility for both of the X-rays and the outer contours of the 3D objects
   ii. Choose *Type* > *X-ray Object Registration*, either of the X-rays as moving entity and one of the 3D objects as fixed entity, then click *OK*
   iii. Now, you should use the navigation buttons in the right viewport to align the selected X-ray, but only use translation, never rotation! Follow the changes in the left viewport!
   iv. When finished, click *Close* in the viewport and repeat the same process, using the other X-ray as moving entity
   v. Between registering the two X-rays, it can help to register the 3D objects (each vertebra)

d. Registration of the 3D objects:
   i. Repeat the registration process for the vertebrae
   ii. In the manual registration dialog, choose *Type* > *Part Registration*
   iii. Now, you are also free to use the rotation functionality
   iv. At the end of this stage, you should have all of the vertebrae nicely aligned with both of the X-ray images (sometimes, this is possible only to a limited extent)

3. Creating contours:
   a. For the next step, we are going to have to draw the contours of all the segments on the X-ray images
   b. Click on *X-RAY* > *Create Contour*
   c. Now, you can draw contours on the X-ray images. One contour can consist of any number of separate lines. When placing the first point, a line will automatically appear and be attracted to the gradient of the X-ray image. If the contour is not correctly attracted to the border of the desired anatomy, CTRL can be held down to disable the attraction and enable the creation of straight contour sections. When CTRL is released, the contour will be attracted again to the gradient. It is also possible to create spline contours while holding SHIFT during point placement. The contour can either be closed by clicking on the first control point or by double-clicking at the desired end location. You can exit the tool (also to start a new curve) by pressing Esc or clicking *X-RAY* > *Create Contour* again.
   d. Only draw contours where the edge of the segment is clear. Better to draw multiple, more precise sections than fewer imprecise ones!

4. Contour-based registration:

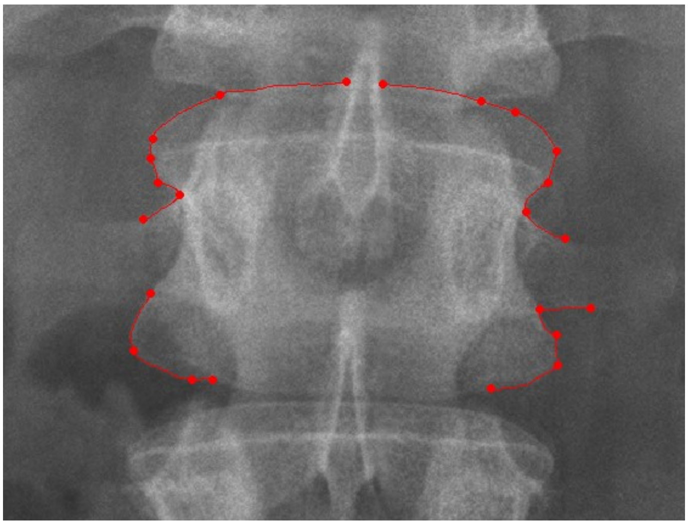

Supp. Figure 6 Contour of a vertebra (Mimics 21.0, materialise.com)

a. Click on *X-RAY > Contour-based Registration*
b. Choose *Type > Part Registration* and select the desired 3D object as moving entity and the coronal and sagittal contours as fixed entities
c. After clicking *OK*, the action takes place

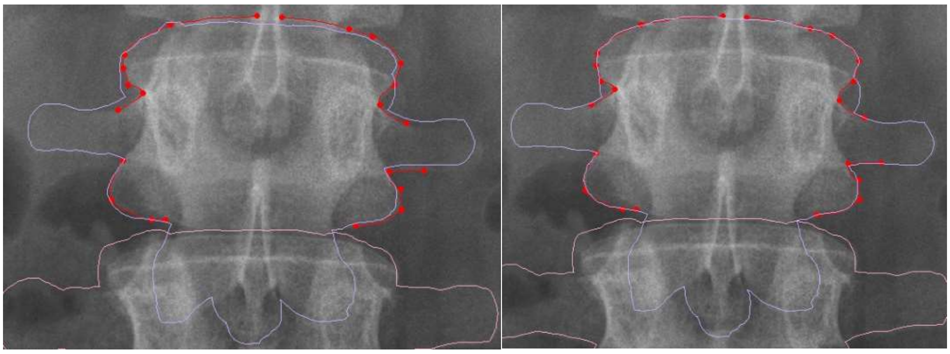

Supp. Figure 7 Before and after contour-based registration (Mimics 21.0, materialise.com)

d. Repeat this procedure for each of the vertebrae with one or both of the X-rays (coronal contour registration yields better results)
e. It is possible, that sometimes the contour-based registration will be seriously off. In those cases, either try to modify the contour for another try or just redo the changes and continue with manual registration.

5. If everything is in order, the spine is now properly registered to the X-ray images. If something is not adding up, try to iterate on the above mentioned steps until everything looks just fine. You can export the newly aligned object as STLs (*File > Export > Binary STL*, select and *Add* the desired 3D objects from the *Part* tab and click *Finish* to save them in your working directory) to make further assesments or just use the bult-in measurement tools.

Supplementary Table 1. Evaluation of the accuracy of the segmentation process

	**DSI** (I_1 vs I_2 segmentation)					
**patient ID**	**L1**	**L2**	**L3**	**L4**	**L5**	**S**
**1**	0.979	0.988	0.999	0.974	0.970	0.982
**2**	0.989	0.989	0.990	0.990	0.987	0.979
**3**	0.991	0.992	0.993	0.990	0.987	0.986
**4**	0.991	0.986	0.988	0.988	0.983	0.973
**5**	0.990	0.991	0.992	0.992	0.990	0.982
**6**	0.991	0.990	0.990	0.991	0.992	0.990
**7**	0.983	0.980	0.973	0.974	0.970	0.980
**8**	0.988	0.990	0.991	0.989	0.987	0.988
**9**	0.967	0.973	0.973	0.972	0.969	0.972
**10**	0.966	0.967	0.973	0.967	0.953	0.978
**11**	0.989	0.988	0.989	0.988	0.978	0.984
**12**	0.990	0.990	0.990	0.984	0.983	0.989
**13**	0.981	0.981	0.981	0.980	0.977	0.978
**14**	0.989	0.991	0.993	0.994	0.991	0.991
**15**	0.989	0.990	0.990	0.989	0.986	0.990
**16**	0.989	0.990	0.992	0.992	0.990	0.990
**17**	0.990	0.992	0.993	0.993	0.992	0.993
**18**	0.993	0.994	0.995	0.994	0.992	0.993
**19**	0.999	1.000	1.000	0.999	0.999	0.981
**20**	0.991	0.990	0.986	0.986	0.975	0.978
**21**	0.992	0.993	0.994	0.993	0.992	0.992
**22**	0.987	0.990	0.992	0.991	0.985	0.987
**23**	0.987	0.984	0.984	0.983	0.980	0.985
**24**	0.986	0.986	0.985	0.983	0.974	0.972
**25**	0.987	0.987	0.990	0.990	0.988	0.987
**26**	0.985	0.988	0.989	0.990	0.988	0.984
**27**	0.989	0.990	0.992	0.990	0.988	0.985
**28**	0.982	0.981	0.982	0.983	0.977	0.983
**29**	0.989	0.989	0.991	0.991	0.988	0.986
**30**	0.989	0.991	0.991	0.991	0.989	0.987
**31**	0.990	0.991	0.991	0.991	0.990	0.988
**32**	0.986	0.988	0.988	0.988	0.981	0.985
**33**	0.987	0.987	0.986	0.986	0.980	0.986
**34**	0.991	0.992	0.993	0.989	0.986	0.983
**35**	0.991	0.992	0.993	0.990	0.986	0.985
**36**	0.992	0.993	0.993	0.993	0.987	0.990
**37**	0.983	0.986	0.986	0.987	0.983	0.987
**38**	0.983	0.985	0.986	0.987	0.983	0.986
**39**	0.960	0.967	0.968	0.965	0.955	0.970
**40**	0.986	0.987	0.987	0.987	0.983	0.986
**41**	0.986	0.988	0.987	0.988	0.986	0.988
**42**	0.985	0.986	0.986	0.986	0.983	0.988
**43**	0.987	0.987	0.988	0.988	0.984	0.985
**44**	0.979	0.983	0.984	0.985	0.984	0.986
**45**	0.924	0.926	0.927	0.929	0.935	0.933
**46**	0.912	0.915	0.919	0.921	0.918	0.938
**47**	0.993	0.993	0.994	0.991	0.989	0.993
**48**	0.992	0.992	0.993	0.993	0.992	0.993
**49**	0.925	0.921	0.924	0.929	0.932	0.937
**50**	0.978	0.977	0.974	0.977	0.976	0.985
**mean**	**0.982**	**0.983**	**0.984**	**0.983**	**0.980**	**0.982**

CT (Computed Tomography), DSI (Dice Similarity Index), I (investigator), L1-5 (lumbar level 1-5), S (sacrum)

Supplementary Table 2. Results for the two-way mixed absolute agreement calculation for ICC

X-ray differences

	ICC	95% Confidence Interval	
		Lower Bound	Upper Bound
inter-rater reliability			
I_1T_1 vs I_2T_1	0.998	0.998	0.999
I_1T_1 vs I_2T_2	0.997	0.996	0.998
I_1T_2 vs I_2T_1	0.990	0.987	0.992
I_1T_2 vs I_2T_2	0.992	0.990	0.994
intra-rater reliability			
I_1T_1 vs I_1T_2	0.991	0.989	0.993
I_2T_1 vs I_2T_2	0.997	0.997	0.998

ICC (intraclass correlation), I (investigator), T (time point)

Supplementary Table 3. Results for the two-way mixed absolute agreement calculation for ICC

CT differences

	ICC	95% Confidence Interval	
		Lower Bound	Upper Bound
inter-rater reliability			
I_1T_1 vs I_2T_1	0.998	0.997	0.998
I_1T_1 vs I_2T_2	0.998	0.998	0.999
I_1T_2 vs I_2T_1	0.993	0.991	0.994
I_1T_2 vs I_2T_2	0.994	0.992	0.995
intra-rater reliability			
I_1T_1 vs I_1T_2	0.994	0.992	0.995
I_2T_1 vs I_2T_2	0.999	0.999	0.999

ICC (intraclass correlation), I (investigator), T (time point)

Supplementary Table 4. Results for the two-way mixed absolute agreement calculation for ICC

Postregistrational midsagittal section differences

	ICC	95% Confidence Interval	
		Lower Bound	Upper Bound
inter-rater reliability			
I_1T_1 vs I_2T_1	0.999	0.998	0.999
I_1T_1 vs I_2T_2	0.996	0.994	0.996
I_1T_2 vs I_2T_1	0.994	0.992	0.995
I_1T_2 vs I_2T_2	0.994	0.992	0.995
intra-rater reliability			
I_1T_1 vs I_1T_2	0.995	0.993	0.996
I_2T_1vsI_2T_2	0.996	0.995	0.997

ICC (intraclass correlation), I (investigator), T (time point)
